
JOURNAL INFORMATION
==============================
NLM Title Abbreviation: J Vet Intern Med
Journal ID: J Vet Intern Med
NLM Journal ID: 8708660
Journal ID: JVIM

Journal of Veterinary Internal Medicine

ISSN: 0891-6640
EISSN: 1939-1676
Publisher: Oxford University Press

ARTICLE INFORMATION
==============================
PMCID: PMC11474531
DOI: 10.1111/jvim.16461
Article ID: JVIM16461
Article version: 1
Subjects: Issue Information, Issue Information

Issue Information

Electronic publication date: 2023 Sep 1
Publication date: 2023 Sep-Oct
Volume: 37
Issue: 5
First page: 1619
Last page: 1625
Copyright: © 2023 The Authors. Journal of Veterinary Internal Medicine published by Wiley Periodicals LLC on behalf of American College of Veterinary Internal Medicine.
License: This is an open access article under the terms of the http://creativecommons.org/licenses/by/4.0/ License, which permits use, distribution and reproduction in any medium, provided the original work is properly cited.
License URL: https://creativecommons.org/licenses/by/4.0/

Figure count: 0
Table count: 0
Page count: 1

source-schema-version-number: 2.0
cover-date: September/October 2023
details-of-publishers-convertor: Converter:WILEY_ML3GV2_TO_JATSPMC version:6.4.9 mode:remove_FC converted:15.10.2024

==============================

Volume 37, Number 5

September/October 2023

### JOURNAL OF VETERINARY INTERNAL MEDICINE

Open Access

ACVIM

American College of Veterinary Internal Medicine

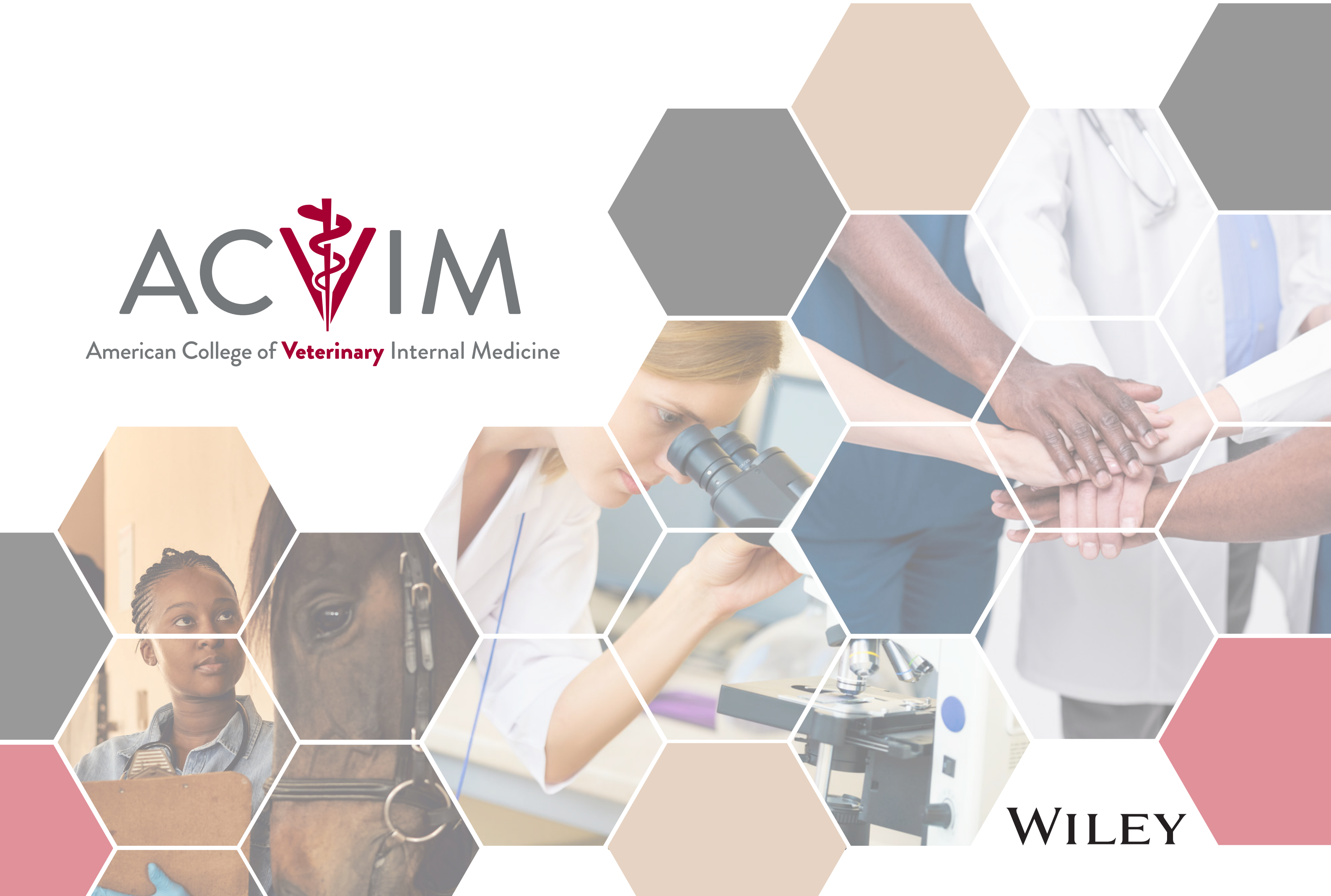

WILEY

The Official Journal of
the American College of Veterinary Internal Medicine,
the European College of Veterinary Internal Medicine -
Companion Animals,
the European College of Veterinary Neurology, and
the European College of Equine Internal Medicine

// Journal of Veterinary Internal Medicine

Open Access

American College of Veterinary Internal Medicine

The Official Journal of the American College of Veterinary Internal Medicine,
the European College of Veterinary Internal Medicine - Companion Animals, the European College of Veterinary Neurology,
and the European College of Equine Internal Medicine

### MISSION STATEMENT

The mission of the *Journal of Veterinary Internal Medicine* is to advance veterinary medical knowledge and improve the lives of animals by publication of authoritative scientific articles of animal diseases.

**Volume 37, Number 5** **September/October 2023**

SMALL ANIMAL

Review Articles

*Endocrinology*

1626 **Influence of medications on thyroid function in dogs: An update**
*Timothy A. Bolton and David L. Panciera*

*Respiratory*

1641 **The respiratory microbiota and its impact on health and disease in dogs and cats: A One Health perspective**
*Aida I. Vientós-Plotts, Aaron C. Ericsson, and Carol R. Reinero*

Standard Articles

*Cardiology*

1656 **Echocardiographic characteristics of dogs with pulmonary hypertension secondary to respiratory diseases**
*Yunosuke Yuchi, Ryohei Suzuki, Takahiro Saito, Yuyo Yasumura, Takahiro Teshima, Hirotaka Matsumoto, and Hidekazu Koyama*

1667 **Investigation of the cardiac effects of exercise testing on apparently healthy Boxer dogs**
*Deepmala Agarwal, Etienne Côté, Lynne O'Sullivan, Kathryn M. Meurs, and Jörg Steiner*

1679 **Systolic third sound associated with systolic anterior motion of the mitral valve in cats with obstructive hypertrophic cardiomyopathy**
*Vittorio Saponaro, Clémence Mey, Irène Vonfeld, Antoine Chamagne, Maria-Paz Alvarado, Jean-luc Cadoré, Valérie Chetboul, and Loïc Desquilbet*

*Endocrinology*

1685 **Prevalence of hypercalcemia in primary hypoadrenocorticism in dogs: Multicenter, retrospective study**
*Harriet Hall, Timothy Williams, Jessica Florey, Mireia Pascual Moreno, Victoria Black, Daniel Thompson, and Barbara Skelly*

1694 **Serum 25-hydroxyvitamin D, vitamin D receptor, and vitamin D binding protein concentrations in dogs with acute pancreatitis compared to healthy control dogs**
*Dohee Lee, Yoonhoi Koo, Yeon Chae, Yeongeun Choi, Taesik Yun, Byeong-Teck Kang, Mhan-Pyo Yang, and Hakhyun Kim*

1703 **Assessment of the FreeStyle Libre 2 interstitial glucose monitor in hypo- and euglycemic cats**
*Alisa S. Berg, Chiquitha D. Crews, Christopher Adin, Adriana Alfonso-Castro, Susan B. Hill, Jocelyn Mott, and Chen Gilor*

*Genetics*

1710 **Novel genetic variant associated with globoid cell leukodystrophy in a family of mixed breed dogs**
*Samantha Hammack, Devon Wallis Hague, Miranda D. Vieson, Elizabeth Esdaile, Shayne S. Hughes, Rebecca R. Bellone, and Annette M. McCoy*

1716 **Precision medicine using whole genome sequencing in a cat identifies a novel COL5A1 variant for classical Ehlers-Danlos syndrome**
*Abigail McElroy, Heather Gray-Edwards, Lyndon M. Coghill, and Leslie A. Lyons*

*Gastroenterology*

1725 **Retrospective evaluation of the etiology and clinical characteristics of peripheral edema in dogs**
*Bradley D. Whelchel, Jean-Sebastien Palerme, Sandy P. Tou, and Jessica L. Ward*

1738 **Association of serum and fecal microRNA profiles in cats with gastrointestinal cancer and chronic inflammatory enteropathy**
*Louise Brogaard, Janne G. Lyngby, Annemarie T. Kristensen, Merete Fredholm, Charlotte R. Bjørnvad, Silke Salavati Schmitz, Ellen Skancke, Joanna S. Morris, Nana Dupont, David Argyle, Armand Sánchez, Anette Spohr, Kasper Graarup-Hansen, Lise N. Nielsen, and Susanna Cirera*

1750 **Fecal acute phase proteins in cats with chronic enteropathies**
*Dimitra A. Karra, Chris C. Chadwick, Evangelia M. Stavroulaki, Matina N. Pitropaki, Evgenia Flouraki, Karin Allenspach, Jonathan A. Lidbury, Joerg M. Steiner, and Panagiotis G. Xenoulis*

*Hepatology*

1760 **Repeated specific canine pancreatic lipase measurements do not identify multiple acquired portosystemic shunts in dogs after extrahepatic portosystemic shunt attenuation**
*Gonçalo Serrano, Nausikaa Devriendt, and Hilde de Rooster*

1766 **Ultrasonographic measurement of gallbladder wall thickness in fasted dogs without signs of hepatobiliary disease**
*Carlos Martinez, Daniel Davies, Séamus Hoey, Robert E. Shiel, and Emma J. O'Neill*

*Infectious Disease*

1772 **Outcomes of treatment of cats with feline infectious peritonitis using parenterally administered remdesivir, with or without transition to orally administered GS-441524**
*Sally J. Coggins, Jacqui M. Norris, Richard Malik, Merran Govendir, Evelyn J. Hall, Benjamin Kimble, and Mary F. Thompson*

1784 **Thirty-two cats with effusive or non-effusive feline infectious peritonitis treated with a combination of remdesivir and GS-441524**
*Jodie Green, Harriet Syme, and Sarah Tayler*

*Nephrology/Urology*

1794 **Effect of storage temperature and time on measurement of serum symmetric dimethylarginine concentration using point-of-care and commercial laboratory analyzers in cats and dogs**
*Marleen Brans, Sofie Marynissen, Femke Mortier, Luc Duchateau, Sylvie Daminet, and Dominique Paepe*

1806 **Pseudomembranous cystitis in cats with presumed or confirmed mineralization: A retrospective study of 26 cases (2016-2021)**
*Olivier Labelle, Dominique Penninck, Emmanuelle M. Butty, Shelly Hahn, and Marilyn Dunn*

*Neurology*

1815 **Diagnosis and management of dogs with degenerative myelopathy: A survey of neurologists and rehabilitation professionals**
*Teryn V. Bouché, Joan R. Coates, Sarah A. Moore, Dominik Faissler, Mark Rishniw, and Natasha J. Olby*

*Nutrition*

1821 **An undernutrition screening score for dogs with protein-losing enteropathy: A prospective multicenter study**
*Florence E. Wootton, Christopher S. F. K. Hoey, Glynn Woods, Silke Salavati Schmitz, Jenny Reeve, Jennifer Larsen, and Aarti Kathrani*

1830 **Changes in diet and supplement use in dogs with cancer**
*Matthew L. Kramer, Jennifer A. Larsen, and Michael S. Kent*

1839 **A blinded, placebo-controlled study on the clinical effects of vitamin E supplementation in dogs with osteoarthritis**
*Casey L. Gordon, Samantha J. Reeves, Richard K. Burchell, Craig Thomson, Arnon Gal, Nicolas Lopez-Villalobos, Natalie S. L. Webster, Kimberley M. Litster, and Richard A. S. Mitchell*

*Oncology*
1848 **A prospective evaluation of the prevalence of thromboemboli and associated hemostatic dysfunction in dogs with carcinoma or sarcoma**
*Paolo Pazzi, Geoffrey T. Fosgate, Anouska Rixon, Josef Hanekom, Annemarie T. Kristensen, and Amelia Goddard*

*Pharmacology*
1864 **Prevalence of antibiotic use for dogs and cats in United States veterinary teaching hospitals, August 2020**
*Amanda L. Beaudoin, Emma R. Bollig, Brandy A. Burgess, Leah A. Cohn, Stephen D. Cole, Jonathan D. Dear, Claire L. Fellman, Erin Frey, Robert Goggs, Andrea Johnston, Amanda J. Kreuder, Kate S. KuKanich, Tessa E. LeCuyer, Julie Menard, Krystle L. Reagan, Jane E. Sykes, Julia K. Veir, Katrina Viviano, Annie Wayne, and Jennifer L. Granick*

Case Report
*Gastroenterology*
1876 **Molnupiravir treatment of 18 cats with feline infectious peritonitis: A case series**
*Okihiro Sase*

**EQUINE**
Standard Articles
*Endocrinology*
1881 **Assessment of the hypothalamic-pituitary-adrenocortical axis function using a vasopressin stimulation test in neonatal foals**
*Erin Elder, David Wong, Katheryn Johnson, Hannah Robertson, Meghan Marner, and Katarzyna Dembek*

*Immunology*
1889 **Transfer of naturally acquired specific passive immunity against *Anaplasma phagocytophilum* in foals in Southeastern Pennsylvania and Northern Maryland**
*Emily K. Rule, Ashley G. Boyle, Darko Stefanovski, Eman Anis, Jennifer Linton, and Olivia Lorello*

*Neurology*
1893 **Evaluation of real-time polymerase chain reaction for the diagnosis of protozoal myeloencephalitis in horses using cerebrospinal fluid**
*Carla Katherine Enriquez, Jennifer K. Morrow, Amy Graves, and Amy Johnson*

*Pharmacology*
1899 **The effects of orally administered trazodone on ambulation and recumbency in healthy horses**
*Kallie Hobbs, Daniela Luethy, Jennifer Davis, Martha Mallicote, Catherine Torcivia, Jeaneen Kulp, Darko Stefanovski, Catherine Futterman, Freya Cooper, and Andrew van Eps*

1907 **Pharmacokinetics and tolerability of multiple-day oral dosing of mycophenolate mofetil in healthy horses**
*Kaitlyn Bello, Gwendolen Lorch, Kyeongmin Kim, Ramiro E. Toribio, Liwei Yan, Zhiliang Xie, Kasey Hill, and Mitch Phelps*

*Respiratory*
1917 **Utility of serum amyloid A in monitoring clinical response to antimicrobial treatment in horses with bacterial pneumonia**
*Kate L. Hepworth-Warren, Krista Estell, Bobby Cowles, Deborah Amodie, and Mark Crisman*

**FOOD ANIMAL**

Standard Articles

*Immunology*

1923 **Comparison of turbidometric immunoassay, refractometry, and gamma-glutamyl transferase to radial immunodiffusion for assessment of transfer of passive immunity in high-risk beef calves**
*Amanda J. Kreuder, Ryan M. Breuer, Caitlin Wiley, Tyler Dohlman, Joseph S. Smith, and Lauren McKeen*

1934 **Comparison of turbidometric immunoassay and brix refractometry to radial immunodiffusion for assessment of colostral immunoglobulin concentration in beef cattle**
*Ryan M. Breuer, Caitlin Wiley, Tyler Dohlman, Joseph S. Smith, Lauren McKeen, and Amanda J. Kreuder*

**ERRATA**

1944 **Correction to "*Escherichia coli*-associated follicular cystitis in dogs: Clinical and pathologic characterization"**

1945 **Erratum for "Incidence of acute kidney injury in dogs with systemic mycotic infections treated with amphotericin B (1996-2020)"**

1946 **Erratum for "Echocardiographic values for normal conditioned and unconditioned North American whippets"**

### Journal of Veterinary Internal Medicine

Open Access

ACVIM American College of Veterinary Internal Medicine

The Official Journal of the American College of Veterinary Internal Medicine,
the European College of Veterinary Internal Medicine - Companion Animals, the European College of Veterinary Neurology,
and the European College of Equine Internal Medicine

### EDITORIAL BOARD

Kenneth W. Hinchcliff, BVSc, PhD, DACVIM
Editor-in-Chief
The University of Melbourne
Stephen P. DiBartola, DVM, DACVIM
Editor-in-Chief
The Ohio State University
Joshua Stern, DVM, PhD, DACVIM
Associate Editor - Cardiology
University of California, Davis
Katharyn Mitchell, BVSc, DipVetClinStud, DVM, PhD, DACVIM
Associate Editor - Large Animal Internal Medicine
(Equine, Cardiology)
Cornell University
Sébastien M. C. Buczinski, DMV, MSc, DACVIM
Consulting Editor for Experimental Design & Statistics
University of Montreal
George Moore, DVM, MS, PhD, DACVIM
Consulting Editor for Experimental Design & Statistics
Purdue University
Kristopher J. Hughes, BVSc (Hons 1), FANZCVS, DECEIM,
Associate ACVIM
ECEIM Associate Editor - Large Animal Internal Medicine (Equine)
Charles Sturt University
Linda Kidd, DVM, PhD, DACVIM
Associate Editor - Small Animal Internal Medicine
(Infectious Disease)
Linda Kidd Veterinary Internal Medicine Consulting
M. Julia B. Felippe, MV, MSc, PhD, DACVIM
Associate Editor - Large Animal Internal Medicine (Equine)
Cornell University
Shelly L. Vaden, DVM, PhD, DACVIM
Associate Editor - Small Animal Internal Medicine
(Nephrology/Urology)
North Carolina State University
Michael Willard, DVM, DACVIM
Associate Editor - Small Animal Internal Medicine
Texas A&M University
Andrea Tipold, DVM, DECVN
ECVN Associate Editor - Neurology
School of Veterinary Medicine Hannover
Robert Rebhun, DVM, PhD, DACVIM
Associate Editor - Hematology/Oncology
University of California, Davis
Eric Zini, PD, PhD, DECVIM-CA
ECVIM-CA Associate Editor - Small Animal Internal Medicine
(Endocrinology)
University of Zurich & Istituto Veterinario di Novara &
University of Padua
Munashe Chigerwe, BVSc, MPH, PhD, DACVIM
Associate Editor - Large Animal Internal Medicine (Food & Fiber)
University of California, Davis
Lauren Trepanier, DVM, PhD, DACVCP, DACVIM
Associate Editor – Small Animal Internal Medicine
(Pharmacology, Pharmacokinetics, Toxicology, and Therapeutic Trials)
University of Wisconsin

**ACVIM Board of Regents Officers**

Jane Sykes, BVSc (Hons), PhD, MBA, DACVIM
Board Chairperson
Joerg Steiner, DMV, PhD, DACVIM, DECVIM-CA
President
Bill Tyrrell, DVM, DACVIM
Treasurer

**Ex-Officio Member of the ACVIM Board of Regents**

Harold McKenzie, DVM, MS, DACVIM
Immediate Past Board Chair

**The Editorial and ACVIM Offices are located at:**

116 Inveress Drive East, Suite 375, Englewood, CO 80112

**Telephone:** 303-231-9933 or 800-245-9081

**Editorial Email:** JVIM@ACVIM.org.
**ACVIM Email:** ACVIM@ACVIM.org.

The Journal is available online at www.wileyonlinelibrary.com/journal/jvim.

**ACVIM Board of Regents Members**

Sandra D. Taylor, DVM, PhD, DACVIM
Large Animal Internal Medicine At Large
Karyn Harrell, DVM, DACVIM
Small Animal Internal Medicine At Large
Phoebe A. Smith, DVM, DACVIM
Private Practice At Large
Roger A. Hostutler, DVM, MS, DACVIM
President, Small Animal Internal Medicine
Chris Sanchez, DVM, PhD, DACVIM
President, Large Animal Internal Medicine
Steven L. Rosenthal, DVM, DACVIM
President, Cardiology
Joan R. Coates, DVM, MS, DACVIM
President, Neurology
Chad Johannes, DVM, DACVIM
President, Oncology
Shannon Carter, EdD, CAE
Chief Executive Officer

### Journal of Veterinary Internal Medicine

Open Access

ACVIM American College of Veterinary Internal Medicine

The Official Journal of the American College of Veterinary Internal Medicine,
the European College of Veterinary Internal Medicine - Companion Animals, the European College of Veterinary Neurology,
and the European College of Equine Internal Medicine

**Disclaimer**
The Publisher, the American College of Veterinary Internal Medicine and Editors cannot be held responsible for errors or any consequences arising from the use of information contained in this journal; the views and opinions expressed do not necessarily reflect those of the Publisher, the American College of Veterinary Internal Medicine or Editors, neither does the publication of advertisements constitute any endorsement by the Publisher, the American College of Veterinary Internal Medicine or Editors of the products advertised.

**Open Access and Copyright**
Articles published by the *Journal of Veterinary Internal Medicine* are fully open access: immediately freely available to read, download and share. *Journal of Veterinary Internal Medicine* articles are published under the terms of the Creative Commons Attribution Non-Commercial License (CC BY NC), which permits use, distribution and reproduction in any medium, provided the original work is properly cited and is not used for commercial purposes.
Copyright on any research article published in the *Journal of Veterinary Internal Medicine* is retained by the author(s). Authors grant Wiley a license to publish the article and identify itself as the original publisher. Authors also grant any third party the right to use the article freely as long as its integrity is maintained and its original authors, citation details and publisher are identified. The Wiley Open Access Agreement formalizes these and other terms and conditions of publishing research articles.

**Use by commercial "for-profit" organizations**
Use of Wiley Open Access articles for commercial, promotional, or marketing purposes requires further explicit permission from Wiley and will be subject to a fee. All requests to republish the article (in whole or in part) in another work such as a book or journal article (other than normal quotations with an appropriate citation) can be cleared directly from the point of content using the automated RightsLink service. Simply click on the 'Request Permission' link to complete your order.
For other commercial, promotional, or marketing purposes please contact corporatesales@wiley.com. Commercial purposes include: Copying or downloading of articles, or linking to such articles for further redistribution, sale or licensing; Copying, downloading or posting by a site or service that incorporates advertising with such content; The inclusion or incorporation of article content in other works or services (for example, a compilation educational purposes; Use for the purposes of monetary reward by means of sale, resale, licence, loan, transfer or other form of commercial exploitation such as marketing products; Print reprints/eprints of Wiley Open Access articles.

*JOURNAL OF VETERINARY INTERNAL MEDICINE* (ISSN 1939-1676 [online]) is published bimonthly on behalf of the American College of Veterinary Internal Medicine by Wiley Periodicals LLC, 111 River St., Hoboken, NJ 07030-5774.

**Publisher**
*Journal of Veterinary Internal Medicine* is published by Wiley Periodicals LLC, 350 Main St., Malden, MA 02148-5020

**Production Editor:** Deepika Prakash (email: JVIM@wiley.com).

**Commercial Reprints:** Helane Daniels (email: hdaniels@wiley.com)

**Abstracting and Indexing Services**
The Journal is indexed by Science Citation Index, and MEDLINE. For a complete list of A&I Services visit: www.wilevonlinelibrarv.com/iournal/jvim

ISSN 1939-1676 (Online)

For submission instructions, and other information visit: www.wileyonlinelibrary.com/journal/jvim

**Manuscript Review**
All manuscripts are reviewed by experts in the field who advise the editors of the manuscript's scientific quality. Decisions regarding publication are made by the Co-Editors-in-Chief acting on the advice of reviewers and Associate Editors.

Wiley's Corporate Citizenship initiative seeks to address the environmental, social, economic, and ethical challenges faced in our business and which are important to our diverse stakeholder groups. Since launching the initiative, we have focused on sharing our content with those in need, enhancing community philanthropy, reducing our carbon impact, creating global guidelines and best practices for paper use, establishing a vendor code of ethics, and engaging our colleagues and other stakeholders in our efforts.

Follow our progress at www.wiley.com/go/citizenship
